# Unintentional injury deaths among children under five in Hunan Province, China, 2015–2020

**DOI:** 10.1038/s41598-023-32401-1

**Published:** 2023-04-04

**Authors:** Xu Zhou, Zhiqun Xie, Jian He, Hong Lin, Juan Xiao, Hua Wang, Junqun Fang, Jie Gao

**Affiliations:** 1 Hunan Provincial Maternal and Child Health Care Hospital, Changsha, 410000 Hunan China; 2 Department of Medical Statistics and Epidemiology, School of Public Health, Sun Yat-Sen University, Guangzhou, 510000 Guangdong China; 3 Department of Medical Genetics, The Hunan Children’s Hospital, Changsha, 410000 Hunan China; 4 National Health Commission Key Laboratory of Birth Defects Research, Prevention and Treatment, Hunan Provincial Maternal and Child Health Care Hospital, Changsha, China

**Keywords:** Medical research, Paediatric research

## Abstract

Injury is the most common cause of preventable morbidity and death among children under five. This study aimed to describe the epidemiological characteristics of injury-related mortality rates in children under five and to provide evidence for future preventive strategies. Data were obtained from the Under Five Child Mortality Surveillance System in Hunan Province, China, 2015–2020. Injury-related mortality rates with 95% confidence intervals (CI) were calculated by year, residence, gender, age, and major injury subtype (drowning, suffocation, traffic injuries, falls, and poisoning). And crude odds ratios (ORs) were calculated to examine the association of epidemiological characteristics with injury-related deaths. The Under Five Child Mortality Surveillance System registered 4,286,087 live births, and a total of 22,686 under-five deaths occurred, including 7586 (which accounted for 33.44% of all under-five deaths) injury-related deaths. The injury-related under-five mortality rate was 1.77‰ (95% CI 1.73–1.81). Injury-related deaths were mainly attributed to drowning (2962 cases, 39.05%), suffocation (2300 cases, 30.32%), traffic injuries (1200 cases, 15.82%), falls (627 cases, 8.27%), and poisoning (156 cases, 2.06%). The mortality rates due to drowning, suffocation, traffic injuries, falls, and poisoning were 0.69‰ (95% CI 0.67,0.72), 0.54‰ (95% CI 0.51,0.56), 0.28‰ (95% CI 0.26,0.30), 0.15‰ (95% CI 0.13,0.16), and 0.04‰ (95% CI 0.03,0.04), respectively. From 2015 and 2020, the injury-related mortality rates were 1.78‰, 1.77‰, 1.60‰, 1.78‰, 1.80‰, and 1.98‰, respectively, and showed an upward trend (*χ*^*2*^_*trend*_ = 7.08, P = 0.01). The injury-related mortality rates were lower in children aged 0–11 months than in those aged 12–59 months (0.52‰ vs. 1.25‰, OR = 0.41, 95% CI 0.39–0.44), lower in urban than rural areas (1.57‰ vs. 1.88‰, OR = 0.84, 95% CI 0.80–0.88), and higher in males than females (2.05‰ vs . 1.45‰, OR = 1.42, 95% CI 1.35–1.49). The number of injury-related deaths decreased with children’s age. Injury-related deaths happened more frequently in cold weather (around February). Almost half (49.79%) of injury-related deaths occurred at home. Most (69.01%) children did not receive treatment after suffering an injury until they died, and most (60.98%) injury-related deaths did not receive treatment because it was too late to get to the hospital. The injury-related mortality rate was relatively high, and we have described its epidemiological characteristics. Several mechanisms have been proposed to explain these phenomena. Our study is of great significance for under-five child injury intervention programs to reduce injury-related deaths.

## Introduction

Injury is a major cause of death of children in China. The overall injury-related under-five mortality rate was 1.6‰ in 2015 in China, and injury is the third leading cause of under-five deaths and the first leading cause of death among children between 1 and 4 years old^1^. Injury is also a major cause of death of children worldwide^2^. The injury-related mortality rate in children is much higher in low- and middle-income countries than in high-income countries, and it is estimated that more than 95% of injury-related deaths in children occur in developing countries^2–5^. There is still much room for a decline in the injury-related under-five mortality rate in China.

There were some studies focused on the epidemiological characteristics of injury-related under-five deaths. E.g., in Turkey (2014–2017), injury-related under-five deaths were mainly attributed to traffic injuries (36.5%), falls (12.0%), and suffocation (10.2%). Of all injury deaths, 59.9% were males, and 52.7% occurred at home or in its close vicinity^6^; In Iran (2010–2015), multivariate logistic regression showed that mothers' low education level, age 1–5 years', living in a supportive center, and having financial problems increased the odds of under-5 mortality caused by injury^7^; In China (2009–2016), injury-related under-five deaths were mainly attributed to suffocation (34.3%), drowning (29.6%), traffic injuries (17.7%), falls (7.2%) or poisoning (4.7%)^8^; From 2013 to 2019 in Sichuan, China, the top three causes of total under-five deaths were accidental drowning (35.0%), accidental suffocation (32.7%), and traffic accidents (15.5%)^9^.

**Figure 1:**
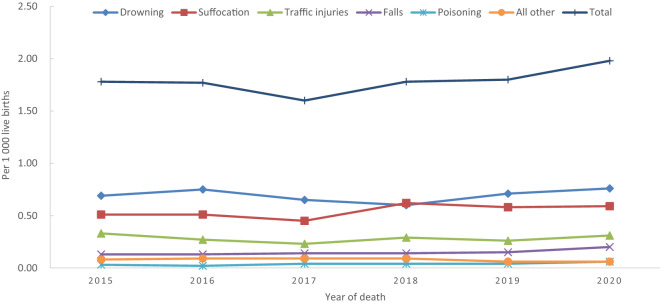
Time trends in injury-related under-five mortality rate in Hunan Province, China, 2015–2020.

Injury is the most common cause of preventable morbidity and mortality among children^10^. Therefore, studies on the epidemiological characteristics of injury-related under-five deaths are important for providing evidence for future intervention^11^. However, the epidemiology of injury-related deaths among children under five has been rarely reported recently, and more studies need to be included in China (Fig. 1).

Therefore, we investigated the epidemiology of the injury-related under-five mortality rate in Hunan Province, China, using Under Five Child Mortality Surveillance System data for the period 2015–2020. The aim of this study was to provide some information for under-five child injury intervention programs to reduce injury-related deaths.

## Methods

### Data sources

This study used data from the Under Five Child Mortality Surveillance System in Hunan Province, China, 2015–2020, which is run by the Hunan Provincial Health Commission and covers all under-five deaths in Hunan Province. Children’s death reports included demographic characteristics such as gender, age, primary cause of death, location of death and other key information. According to the WHO International Classification of Diseases (Tenth Revision, ICD-10), 23 injuries in this study were classified into five types: drowning (W65–W74), traffic injury (V01–V98), suffocation (W75–W84), poisoning (X44–X49), fall (W00–W19), or “other” (W20–W64, W85–W94, X00–X43, X50–X59).

Our data were derived from the Under Five Child Mortality Surveillance System. It is the second use of the data, and no further ethical approval was required for the present study.

### Data quality control

To carry out surveillance, the Hunan Provincial Health Commission formulated the "Maternal and Child Health Monitoring Manual in Hunan Province". Data were collected and reported by experienced doctors. To reduce the integrity rate and information error rate, we asked the technical guidance departments to carry out comprehensive quality control each year.

### Statistical analysis

The injury-related mortality rate is defined as the number of deaths from injury per 1000 live births (‰). We calculated the injury-related mortality rate and 95% confidence intervals (CI) by Poisson’s regression. Chi-square trend tests (χ^2^_trend_) were used to determine trends in mortality rates by year. Crude odds ratios (ORs) were calculated to examine the association of each epidemiological characteristic with injury-related deaths.

All statistical analyses in this study were performed using SPSS 18.0 (International Business Machines Corporation, New York City, United States).

## Results

### Injury-related under-five mortality rate in Hunan Province, China, 2015–2020

Our study included 4,286,087 live births, and a total of 22,686 under-five deaths occurred, including 7586 (accounted for 33.44% of all under-five deaths) injury-related deaths. The injury-related under-five mortality rate was 1.77‰ (95% CI 1.73–1.81). Table 1 shows the injury-related mortality rates and proportions by year, residence, gender, and age. (Table 1).

**Table 1:** Injury-related under-five mortality rate in Hunan Province, China, 2015–2020.

Characteristic of injury death	Number of live births (n)	Injury-related under-five deaths (n)	Injury-related under-five mortality rate (‰, 95% CI)	Total under-five deaths (n)	Proportion of injury-related deaths in total under-five deaths (%)
Year					
2015	781,066	1387	1.78 (1.68–1.87)	4795	28.93
2016	795,399	1404	1.77 (1.67–1.86)	4435	31.66
2017	834,955	1338	1.60 (1.52–1.69)	4084	32.76
2018	705,524	1253	1.78 (1.68–1.87)	3766	33.27
2019	632,461	1141	1.80 (1.70–1.91)	3207	35.58
2020	536,682	1063	1.98 (1.86–2.10)	2399	44.31
Residence					
Urban	1,571,648	2475	1.57 (1.51–1.64)	7514	32.94
Rural	2,714,439	5111	1.88 (1.83–1.93)	15,172	33.69
Gender					
Male	2,269,408	4663	2.05 (2.00–2.11)	13,201	35.32
Female	2,016,646	2922	1.45 (1.40–1.50)	9467	30.87
Unknown	33	1	–	18	5.56
Age (months)					
0–11	4,286,087	2223	0.52 (0.50–0.54)	14,158	15.70
12–59	4,286,087	5363	1.25 (1.22–1.28)	8528	62.89
Total	4,286,087	7586	1.77 (1.73–1.81)	22,686	33.44

*CI* confidence interval.

### Injury-related under-five mortality rate by injury type

Injury-related deaths were mainly attributed to drowning (2962 cases, 39.05%), suffocation (2300 cases, 30.32%), traffic injuries (1200 cases, 15.82%), falls (627 cases, 8.27%), and poisoning (156 cases, 2.06%). The mortality rates due to drowning, suffocation, traffic injuries, falls, and poisoning were 0.69‰ (95% CI 0.67, 0.72), 0.54‰ (95% CI 0.51, 0.56), 0.28‰ (95% CI 0.26, 0.30), 0.15‰ (95% CI 0.13, 0.16), and 0.04‰ (95% CI 0.03, 0.04), respectively. (Table 2).

**Table 2:** Injury-related under-five mortality rate by injury type.

Injury type	Number of live births (n)	Number of deaths (n)	Mortality rate (‰, 95% CI)	Proportion in total injury-related deaths (%)
Drowning	4,286,087	2962	0.69(0.67–0.72)	39.05
Suffocation	4,286,087	2300	0.54(0.51–0.56)	30.32
Traffic injuries	4,286,087	1200	0.28(0.26–0.30)	15.82
Falls	4,286,087	627	0.15(0.13–0.16)	8.27
Poisoning	4,286,087	156	0.04(0.03–0.04)	2.06
All other	4,286,087	341	0.08(0.07–0.09)	4.50
Total	4,286,087	7586	1.77(1.73–1.81)	100.00

*CI* confidence interval.

### Injury-related under-five mortality rate by year

From 2015 and 2020, the injury-related mortality rates were 1.78‰, 1.77‰, 1.60‰, 1.78‰, 1.80‰, and 1.98‰, respectively, and showed an upward trend (χ^2^_trend_ = 7.08, P = 0.01). The mortality rates from suffocation, falls, and poisoning showed increasing trends, too (P < 0.05). (Table 3).

**Table 3:** Injury-related under-five mortality rate by year.

Injury type	2015 (n, ‰) (N = 781,066)	2016 (n, ‰) (N = 795,399)	2017 (n, ‰) (N = 834,955)	2018 (n, ‰) (N = 705,524)	2019 (n, ‰) (N = 632,461)	2020 (n, ‰) (N = 536,682)	*χ*^*2*^_*trend*_	*P*
Drowning	541 (0.69)	596 (0.75)	542 (0.65)	424 (0.60)	450 (0.71)	409 (0.76)	0.10	0.76
Suffocation	397 (0.51)	402 (0.51)	376 (0.45)	437 (0.62)	369 (0.58)	319 (0.59)	12.54	0.00
Traffic injuries	260 (0.33)	212 (0.27)	194 (0.23)	205 (0.29)	164 (0.26)	165 (0.31)	0.66	0.42
Falls	103 (0.13)	104 (0.13)	119 (0.14)	98 (0.14)	95 (0.15)	108 (0.20)	8.90	0.00
Poisoning	23 (0.03)	19 (0.02)	30 (0.04)	28 (0.04)	25 (0.04)	31 (0.06)	8.93	0.00
All other	63 (0.08)	71 (0.09)	77 (0.09)	61 (0.09)	38 (0.06)	31 (0.06)	4.47	0.03
Total	1387 (1.78)	1404 (1.77)	1338 (1.60)	1253 (1.78)	1141 (1.80)	1063 (1.98)	7.08	0.01

*N* number of live births.

### Injury-related under-five mortality rate by age

The injury-related mortality rates were lower in children aged 0–11 months than in those aged 12–59 months (0.52‰ vs. 1.25‰, OR = 0.41, 95% CI 0.39–0.44). Injury-related deaths due to drowning (OR = 0.02, 95% CI 0.02–0.03), traffic injuries (OR = 0.10, 95% CI 0.08–0.12), falls (OR = 0.18, 95% CI 0.14–0.22), and poisoning (OR = 0.27, 95% CI 0.18–0.39) were more common in children aged 12–59 months, while deaths due to suffocation were more common in children aged 0–11 months (OR = 4.03, 95% CI 3.64–4.47). (Table 4).

**Table 4:** Injury-related under-five mortality rate by age.

Injury type	Injury-related deaths of children aged 0–11 months (N = 4,286,087)		Injury-related deaths of children aged 12–59 months (N = 4,286,087) (Reference)		OR (95% CI)
	n	Mortality rate (‰, 95% CI)	n	Mortality rate (‰, 95% CI)	
Drowning	57	0.01 (0.01–0.02)	2905	0.68 (0.65–0.70)	0.02 (0.02–0.03)
Suffocation	1843	0.43 (0.41–0.45)	457	0.11 (0.10–0.12)	4.03 (3.64–4.47)
Traffic injuries	111	0.03 (0.02–0.03)	1089	0.25 (0.24–0.27)	0.10 (0.08–0.12)
Falls	95	0.02 (0.02–0.03)	532	0.12 (0.11–0.13)	0.18 (0.14–0.22)
Poisoning	33	0.01 (0.05–0.11)	123	0.03 (0.02–0.03)	0.27 (0.18–0.39)
All other	84	0.02 (0.02–0.02)	257	0.06 (0.05–0.07)	0.33 (0.26–0.42)
Total	2223	0.52 (0.50–0.54)	5363	1.25 (1.22–1.28)	0.41 (0.39–0.44)

*N* number of live births, *OR* crude odds ratio, *CI* confidence interval.

### Injury-related under-five mortality rate by residence

The injury-related mortality rates were lower in urban than rural areas (1.57‰ vs. 1.88‰, OR = 0.84, 95% CI 0.80–0.88). Injury-related deaths due to drowning (OR = 0.89, 95% CI 0.82–0.96), suffocation (OR = 0.79, 95% CI 0.73–0.87), and traffic injuries (OR = 0.81, 95% CI 0.72–0.92) were more common in rural areas. (Table 5).

**Table 5:** Injury-related under-five mortality rate by residence.

Injury type	Urban (N = 1,571,648)		Rural (N = 2,714,439) (Reference)		OR (95% CI)
	n	Mortality rate (‰, 95% CI)	n	Mortality rate (‰, 95% CI)	
Drowning	1004	0.64 (0.60–0.68)	1958	0.72 (0.69–0.75)	0.89 (0.82–0.96)
Suffocation	724	0.46 (0.43–0.49)	1576	0.58 (0.55–0.61)	0.79 (0.73–0.87)
Traffic injuries	384	0.24 (0.22–0.27)	816	0.30 (0.28–0.32)	0.81 (0.72–0.92)
Falls	212	0.14 (0.12–0.15)	415	0.15 (0.14–0.17)	0.88 (0.75–1.04)
Poisoning	54	0.03 (0.03–0.04)	102	0.04 (0.03–0.05)	0.91 (0.66–1.27)
All other	97	0.06 (0.05–0.07)	244	0.09 (0.08–0.10)	0.69 (0.54–0.87)
Total	2475	1.57 (1.51–1.64)	5111	1.88 (1.83–1.93)	0.84 (0.80–0.88)

*N* number of live births, *OR* crude odds ratio, *CI* confidence interval.

### Injury-related under-five mortality rate by gender

The injury-related mortality rates were higher in males than females (2.05‰ vs. 1.45‰, OR = 1.42, 95% CI 1.35–1.49). Injury-related deaths due to drowning (OR = 1.80, 95% CI 1.66–1.94), suffocation (OR = 1.22, 95% CI 1.12–1.33), traffic injuries (OR = 1.13, 95% CI 1.01–1.27), and falls (OR = 1.35, 95% CI 1.15–1.58) were more common in males. (Table 6).

**Table 6:** Injury-related under-five mortality rate by gender.

Injury type	Male (N = 2,269,408)		Female (N = 2,016,646) (**Reference**)		OR (95% CI)
	n	Mortality rate (‰, 95% CI)	n	Mortality rate (‰, 95% CI)	
Drowning	1981	0.87 (0.83–0.91)	981	0.49 (0.46–0.52)	1.80 (1.66–1.94)
Suffocation	1331	0.59 (0.56–0.62)	968	0.48 (0.45–0.51)	1.22 (1.12–1.33)
Traffic injuries	673	0.30 (0.27–0.32)	527	0.26 (0.24–0.28)	1.13 (1.01–1.27)
Falls	378	0.17 (0.15–0.18)	249	0.12 (0.11–0.14)	1.35 (1.15–1.58)
Poisoning	92	0.04 (0.03–0.05)	64	0.03 (0.02–0.04)	1.28 (0.93–1.76)
All other	208	0.09 (0.08–0.10)	133	0.07 (0.06–0.08)	1.39 (1.12–1.73)
Total	4663	2.05 (2.00–2.11)	2922	1.45 (1.40–1.50)	1.42 (1.35–1.49)

*N* number of live births, *OR* crude odds ratio, *CI* confidence interval.

### Proportions of injury-related under-five deaths by injury types and epidemiological characteristics

Table 7 showed the following epidemiological characteristics of injury-related deaths: (1) The number of injury-related deaths decreased with children’s age. (2) Injury-related deaths happened more frequently in cold weather (around February). (3) Almost half (49.79%) of injury-related deaths occurred at home. (4) Most (69.01%) children did not receive treatment after suffering an injury until they died, and most (60.98%) injury-related deaths did not receive treatment because it was too late to get to the hospital.

**Table 7:** Proportions of injury-related under-five deaths by injury types and epidemiological characteristics.

Basic information	Drowning	%	Suffocation	%	Traffic injuries	%	Falls	%	Poisoning	%	All other	%	Total	%
Age (months)														
0–11	57	1.92	1843	80.13	111	9.25	95	15.15	33	21.15	84	24.63	2223	29.30
12–23	1112	37.54	214	9.30	303	25.25	125	19.94	42	26.92	70	20.53	1866	24.60
24–35	1019	34.40	116	5.04	289	24.08	131	20.89	23	14.74	84	24.63	1662	21.91
36–47	487	16.44	70	3.04	274	22.83	156	24.88	34	21.79	57	16.72	1078	14.21
48–59	287	9.69	57	2.48	223	18.58	120	19.14	24	15.38	46	13.49	757	9.98
Month of death														
January	212	7.16	370	16.09	84	7.00	49	7.81	31	19.87	46	13.49	792	10.44
February	219	7.39	365	15.87	128	10.67	57	9.09	23	14.74	35	10.26	827	10.90
March	276	9.32	251	10.91	95	7.92	69	11.00	10	6.41	36	10.56	737	9.72
April	262	8.85	194	8.43	107	8.92	53	8.45	12	7.69	19	5.57	647	8.53
May	243	8.20	137	5.96	102	8.50	47	7.50	7	4.49	29	8.50	565	7.45
June	257	8.68	108	4.70	104	8.67	48	7.66	11	7.05	31	9.09	559	7.37
July	309	10.43	87	3.78	103	8.58	50	7.97	5	3.21	30	8.80	584	7.70
August	296	9.99	76	3.30	103	8.58	60	9.57	8	5.13	13	3.81	556	7.33
September	200	6.75	85	3.70	94	7.83	52	8.29	4	2.56	22	6.45	457	6.02
October	252	8.51	138	6.00	106	8.83	50	7.97	9	5.77	29	8.50	584	7.70
November	222	7.49	190	8.26	79	6.58	44	7.02	14	8.97	28	8.21	577	7.61
December	214	7.22	299	13.00	95	7.92	48	7.66	22	14.10	23	6.74	701	9.24
Places of death														
Home	1806	60.97	1371	59.61	180	15.00	186	29.67	74	47.44	160	46.92	3777	49.79
Hospital	340	11.48	563	24.48	409	34.08	293	46.73	62	39.74	111	32.55	1778	23.44
On the way to the hospital	528	17.83	324	14.09	499	41.58	117	18.66	16	10.26	55	16.13	1539	20.29
On the way home after hospital transfer or treatment	5	0.17	20	0.87	9	0.75	8	1.28	3	1.92	3	0.88	48	0.63
Other	253	8.54	6	0.26	85	7.08	18	2.87	0	0.00	8	2.35	370	4.88
Missing value	30	1.01	16	0.70	18	1.50	5	0.80	1	0.64	4	1.17	74	0.98
Die before treatment														
No treatment	2437	82.28	1552	67.48	702	58.50	265	42.26	85	54.49	194	56.89	5235	69.01
Outpatient	414	13.98	487	21.17	287	23.92	169	26.95	36	23.08	67	19.65	1460	19.25
Hospitalisation	61	2.06	230	10.00	192	16.00	182	29.03	34	21.79	71	20.82	770	10.15
Other	50	1.69	31	1.35	19	1.58	11	1.75	1	0.64	9	2.64	121	1.60
Reasons for lack of treatment														
Too late to go to the hospital	2135	72.08	1376	59.83	651	54.25	232	37.00	76	48.72	156	45.75	4626	60.98
Parents think it was unserious	1	0.03	14	0.61	1	0.08	3	0.48	2	1.28	4	1.17	25	0.33
Traffic inconvenience	0	0.00	9	0.39	0	0.00	4	0.64	1	0.64	0	0.00	14	0.18
Economic difficulties	0	0.00	5	0.22	2	0.17	0	0.00	1	0.64	1	0.29	9	0.12
Other reasons or treatment	826	27.89	896	38.96	546	45.50	388	61.88	76	48.72	180	52.79	2912	38.39
Total	2962	39.05	2300	30.32	1200	15.82	627	8.27	156	2.06	341	4.50	7586	100.00

The epidemiological characteristics of some injury subtypes differed from those described above: (1) Most (71.94%) deaths due to drowning occurred in children aged 12–35 months, and were more common in July. (2) Most (80.13%) deaths due to suffocation occurred in children aged 0–11 months, and were more common in January. (3) Most deaths due to traffic injuries happened on the way to the hospital (41.58%) and in the hospital (34.08%) (Table 7).

## Discussion

Overall, we found that drowning and suffocation were the most common injury types for under-five deaths. Injury-related under-five deaths were more common in children aged 12–59 months, those living in rural areas, and males. In addition, we found that injury-related under-five deaths were associated with some epidemiological characteristics.

The overall injury-related under-five mortality rate (1.77‰) in our study is consistent with the reported mortality rate in China (1.6‰ in 2015)^1^. Several comprehensive studies showed that the injury-related under-five mortality rate in China is higher than in developed countries^2,4^. The following are injury-related under-five mortality rates reported in some regions: 9.1/100,000 in Turkey in 2017^6^; 39.5/100,000 in Pakistan, 2006–2007^12^; 302/100,000 in India in 2005^13^; 48.96/10,000 in Hunan Province, China, 2009–2014^14^; 1.7–3.8‰ in Sichuan Province, China, 2009–2017^15^. There are major differences between them. It may indicate that there are large geographical differences in injury-related under-five mortality rates, and that injury-related under-five mortality rates are changing over time. In this study, the proportion of injury-related under-five deaths in total under-five deaths increased steadily from 2015 to 2020, and the injury-related under-five mortality rate showed an upward trend, while some previous studies showed downward trends^15,16^. It suggested that injuries were gradually becoming the leading cause of under-five deaths in Hunan Province, China. Therefore, it deserves our special attention.

Previous studies showed that the leading injury types worldwide for under-five deaths were traffic injuries, drowning, burn, suffocation, and falls^4^. Chen et al. reported that the leading injury types for under-five deaths were traffic accidents (57.44%) and drowning (35.53%) in China, 2006–2017^16^. It is different from our study. And many other studies also reported different injury types for under-five deaths^14,15,17–22^. It indicated that there are differences in injury types for under-five deaths in different areas, and also that the leading injury types for under-five deaths are changing over time.

In this study, drowning was the leading cause of injury-related under-five deaths, which may be mainly related to open bodies of water, such as rivers and lakes. And it is more common in rural areas. It is consistent with some previous studies^9,23–25^. For most open bodies of water in rural areas, there were no protective measures and little reliable adult supervision for children. And it may increase the risk of drowning. Suffocation was the second-leading cause of injury-related under-five deaths and the leading cause of injury-related deaths for children aged 0–11 months. It is consistent with previous studies^9^. We have looked at the causes of suffocation and found most deaths due to suffocation occur in bed and through the inhalation of milk. It is consistent with previous studies^26,27^. It may be mainly related to the negligence and poor first-aid knowledge of caregivers^27–29^. From 2015 to 2020, the injury-related mortality rate due to suffocation increased year over year, while Wang et al. reported a steady suffocation mortality rate among children under five in China, 2006–2016^28^. One possible explanation is China’s two-child policy since 2014^30^, which increases caregivers' burden. It may require further, in-depth research.

Overall, we found injury-related under-five mortality rates were higher in children aged 12–59 months than those aged 0–11 months, higher in rural than urban areas, and higher in males than females. It is consistent with previous studies^15,23–25,31–36^. Although the injury-related mortality rate was higher in children aged 12–59 months than those aged 0–11 months, the number of injury-related deaths decreased with age by year. It is consistent with previous studies^37^. It may be associated with the poor self-protection ability of young children^24^. Higher injury-related mortality rates in rural areas may be related to the poor living environment, residents' safety literacy, and first-aid ability^32,38–40^. Higher injury-related mortality rates in males may be related to differences in biological temperament, cognitive strategies, exposure opportunity, and gender socialization^15^.

However, some injury subtypes were inconsistent with the overall injuries in terms of epidemiology. e.g., the mortality rate due to suffocation was higher in children aged 0–11 months than in those aged 12–59 months. Most deaths due to suffocation occurred in bed or through the inhalation of milk, as mentioned in the previous paragraph. It may be mainly related to the negligence and poor first-aid knowledge of caregivers. There were no statistically significant differences in mortality rates from falls and poisoning between urban and rural areas or in the mortality rate due to poisoning between males and females. It has been less well reported. Brito et al. found that use of the high net, the presence of stairs or steps without a handrail, and exits and passages kept with toys, furniture, boxes, or other items that may be obstructive were associated with the risk of falls in children under five years of age^41^. Children’s falls and poisoning injuries are more often the result of accidents than suicide.

In addition, some other epidemiological characteristics are associated with the injury-related under-five mortality rate. And some of them were rarely covered in previous studies. E.g., total injury deaths happened more often in cold weather (around February), while drowning deaths were more common in July. It is consistent with previous studies^9,42,43^. It is related to several factors: first, bad weather and poor road conditions in cold weather increase the risks of traffic accidents, and a large number of people travel to celebrate the Chinese Spring Festival (mostly celebrated in February), which also increases the risks of traffic accidents; Second, people take measures to keep warm in cold weather, which increases the risks of suffocation and gas poisoning; Third, higher temperatures in July increase the risks of children's exposure to water and drowning, and summer vacations in July and August also increase the risks of children's exposure to water and drowning. Most deaths due to drowning, suffocation, and poisoning occur at home. It is the result of most injuries occurring suddenly at home, and children receive no treatment until they die because it is too late to go to the hospital^44^. Instead, most deaths due to traffic injuries and falls occurred in hospitals or on the way to hospitals. It is the result of the fact that most traffic injuries and falls do not cause immediate deaths, leading to a large number of outpatient and hospitalized patients.

Some things could be improved in our study. As such, we did not analyze some epidemiological features due to data limitations, including economic conditions and educational levels of children’s parents.

## Conclusion

In summary, our data indicated that the injury-related mortality rate was relatively high, and we have described its epidemiological characteristics. Several mechanisms have been proposed to explain these phenomena. Our study is of great significance for under-five child injury intervention programs to reduce injury-related deaths.

## Data Availability

All data generated or analysed during this study are included in this published article.
